# Increased STAT1 Signaling in Endocrine-Resistant Breast Cancer

**DOI:** 10.1371/journal.pone.0094226

**Published:** 2014-04-11

**Authors:** Rui Huang, Dana Faratian, Andrew H. Sims, Danielle Wilson, Jeremy S. Thomas, David J. Harrison, Simon P. Langdon

**Affiliations:** 1 Institute of Genetics and Molecular Medicine, University of Edinburgh, Edinburgh, Scotland, United Kingdom; 2 Department of Pathology, Western General Hospital, Edinburgh, Scotland, United Kingdom; 3 Pathology, Medical and Biological Sciences Building, University of St Andrews, North Haugh, St. Andrews, Fife, Scotland, United Kingdom; Texas A&M University, United States of America

## Abstract

Proteomic profiling of the estrogen/tamoxifen-sensitive MCF-7 cell line and its partially sensitive (MCF-7/LCC1) and fully resistant (MCF-7/LCC9) variants was performed to identify modifiers of endocrine sensitivity in breast cancer. Analysis of the expression of 120 paired phosphorylated and non-phosphorylated epitopes in key oncogenic and tumor suppressor pathways revealed that STAT1 and several phosphorylated epitopes (phospho-STAT1(Tyr701) and phospho-STAT3(Ser727)) were differentially expressed between endocrine resistant and parental controls, confirmed by qRT-PCR and western blotting. The STAT1 inhibitor EGCG was a more effective inhibitor of the endocrine resistant MCF-7/LCC1 and MCF-7/LCC9 lines than parental MCF-7 cells, while STAT3 inhibitors Stattic and WP1066 were equally effective in endocrine-resistant and parental lines. The effects of the STAT inhibitors were additive, rather than synergistic, when tested in combination with tamoxifen *in vitro*. Expression of STAT1 and STAT3 were measured by quantitative immunofluorescence in invasive breast cancers and matched lymph nodes. When lymph node expression was compared to its paired primary breast cancer expression, there was greater expression of cytoplasmic STAT1 (∼3.1 fold), phospho-STAT3(Ser727) (∼1.8 fold), and STAT5 (∼1.5 fold) and nuclear phospho-STAT3(Ser727) (∼1.5 fold) in the nodes. Expression levels of STAT1 and STAT3 transcript were analysed in 550 breast cancers from publicly available gene expression datasets (GSE2990, GSE12093, GSE6532). When treatment with tamoxifen was considered, STAT1 gene expression was nearly predictive of distant metastasis-free survival (DMFS, log-rank p = 0.067), while STAT3 gene expression was predictive of DMFS (log-rank p<0.0001). Analysis of STAT1 and STAT3 protein expression in a series of 546 breast cancers also indicated that high expression of STAT3 protein was associated with improved survival (DMFS, p = 0.006). These results suggest that STAT signaling is important in endocrine resistance, and that STAT inhibitors may represent potential therapies in breast cancer, even in the resistant setting.

## Introduction

The STAT (Signal Transducer and Activator of Transcription) family of proteins mediate cytokine and growth factor receptor signaling, which in turn regulate cell growth, survival, and differentiation [1]–[5]. To date, seven STAT proteins have been identified: STAT1, 2, 3, 4, 5a, 5b, and 6 [1]–[5]. STAT is activated by binding of the STAT molecule to activated receptors through the SH2 domain, which permits dimerization of STATs and their translocation into the nucleus to regulate downstream genes [1]–[5]. It is widely agreed that tyrosine phosphorylation (at a site near residue 700) is required for STAT protein activation and dimerization and this is activated further by serine phosphorylation at a site near residue 725 [1]–[5].

While all seven STAT-family members have been shown to be expressed in breast cancer cell lines, only STATs 1, 3, 5a, and 5b are expressed in breast cancer tissues [6], [7]. A number of studies have implicated both oncogenic and tumor suppressor functions for STAT family members in breast cancer and it seems likely that individual STAT isoforms have pleiotropic functions at different stages of disease progression [6], [7]. At initiation, STAT3 and STAT5 are generally considered to be oncogenic while STAT1 is considered to have a tumor suppressor role [8], [9]. STAT3 and STAT5 have both been implicated in endocrine resistance [10], particularly in growth factor-stimulated disease, but little is known of the role of STAT1 in hormonal control. STAT1 and STAT3 activation are frequently reciprocally regulated and perturbation of their balanced expression or phosphorylation may re-direct cytokine/growth factor signals from proliferative to apoptotic, or from inflammatory to anti-inflammatory [11]. The roles of STAT1 and STAT3 in breast cancer remain controversial since multiple studies have reported variable results between STAT isoform expression and clinical outcome, suggesting a degree of complexity in STAT signaling which is poorly understood. For example, STAT1 expression has been associated with poor outcome [12] while increased phospho-STAT1(Tyr701) expression has been associated with both poor [13] and favourable survival [14]. Data for STAT3 are similarly variable. Several reports describes both increased total and phospho-STAT3(Tyr705) being associated with improved survival [15], [16], but these observations contrast with reports of increased phospho-STAT3(Tyr705) [17] or total STAT3 being associated with poor survival [18]. Reduced activation of STAT3 has been identified after tamoxifen treatment in estrogen receptor alpha (ER) positive tumors [19], indicating a possible connection between reduced STAT3 activity and sensitivity to tamoxifen, and the prospect of enhancement of STAT3 pathway activation in tamoxifen resistant tumors [10].

In a search for pathways that might influence endocrine therapy sensitivity and resistance, we initially carried out an unsupervised interrogation of biochemical signaling pathways using phospho-protein antibody arrays. STAT1, phospho-STAT1(Tyr701), and phospho-STAT3(Ser727) were differentially expressed in endocrine resistant cell lines relative to parental cells. STAT1 expression is known to be higher in the luminal (ER-positive) molecular subtype of breast cancer relative to HER2-positive or triple-negative breast disease [20], but we are not aware of any reports of its role in endocrine-resistant breast cancer. This study aimed to investigate further the roles of STAT1 and STAT3 in endocrine sensitive and resistant breast cancer using both cell line models and clinical samples from breast cancer patients.

## Materials and Methods

### Cell lines and culture conditions

MCF-7 cells were cultured in DMEM (phenol red positive, Gibco) supplemented with 10% heat-inactivated foetal calf serum (FCS) and penicillin/streptomycin (100 IU/mL) in a humidified atmosphere of 5% CO_2_ at 37°C. MCF-7/LCC1 [21] and MCF-7/LCC9 cells [22] were grown in DMEM (phenol red free, Gibco) supplemented with 5% double charcoal-stripped serum, glutamine (0.3 mg/mL), and penicillin/streptomycin (100 IU/mL) in a humidified atmosphere of 5% CO_2_ at 37°C.

### Cell viability analysis by Sulforhodamine B (SRB) assay

Cells were harvested in log phase, then seeded into 96-well cell culture plates. MCF-7 cells were washed with PBS and then transferred to phenol red-free DMEM containing double charcoal-stripped serum after 48 h, and incubated for a further 48 h. MCF-7/LCC1 and MCF-7/LCC9 cells were maintained in double charcoal-stripped medium for 96 h. Cells were then treated with STAT inhibitors. The STAT1 inhibitor (-)-epigallocatechin gallate (EGCG) is a major component of green tea and was obtained from Sigma Aldrich [23]. The STAT3 inhibitors Stattic [24], [25] and WP1066 [26] were both obtained from Calbiochem. Stattic is a non-peptide small molecule inhibitor reported to inhibit STAT3 dimerization by selectively interacting with the STAT3 SH2 domain [24]. WP1066 has been shown to inhibit STAT3 signal pathway by down-regulating STAT3 targets and activating Bax to inhibit STAT3 nuclear localization [26]. Recent data has suggested that Stattic may also interact with STAT1, so it is feasible that some of its action may be mediated via STAT1 [27]. After incubation for 5 days with inhibitors, cells were fixed using 25% cold trichloroacetic acid (Sigma), and incubated for 1 h at 4°C. Plates then were washed, air-dried, and stained with sulforhodamine B (Sigma) dye (0.4% solution in 1% acetic acid). After being washed with 1% acetic acid, Tris buffer (10 mM, pH 10.5) was added to each well 1 h prior to the optical density (OD) being read using a Biohit BP800 Microplate reader at 540 nm.

### Protein expression analysis by western blotting

50 ug of protein lysate were electrophoretically resolved on 10% SDS-PAGE and transferred overnight onto nitrocellulose membranes (Millipore). After transfer, membranes were blocked with LiCor Odyssey Blocking Buffer for 1 h before probing with the appropriate primary antibody overnight at 4°C. Primary antibodies used for western blotting were as follows: phospho-STAT1 (Tyr 701) (Cell Signaling), STAT1, phospho-STAT3 (Ser727), STAT3 (all from Eurogentec). All primary antibodies were used at 1∶1000 dilution. The membranes were then incubated with fluorescently-labelled secondary antibodies diluted with Odyssey Blocking Buffer and then scanned on a Li-Cor Odyssey scanner, and the fluorescence value (integrated intensity, I.I.) corresponded with the detected protein expression levels. An α-tubulin (Abcam) antibody was used as a loading control.

### Cell line RNA extraction and two step real-time PCR

Total RNA was extracted from cultured cells using the Qiagen Mini RNeasy Kit. The concentration and quality of RNA was assessed using the Agilent RNA 6000 Nano kit on the Agilent bioanalyser (Agilent Technologies). 1 µg of total RNA from each individual sample was reverse transcribed using the QuantiTect Reverse Transcription kit (Qiagen) following the manufacturer's protocol. 1 µg total RNA produced 20 µL of cDNA following reverse transcription. cDNA was quantified using the Rotorgene (Corbett Research, San Francisco, CA) and the QuantiTect SYBR Green system (Qiagen) following the manufacturers' instructions. The primers for STAT1, STAT3, and β-actin (house-keeping gene) were obtained from Qiagen. The PCR protocol used was: initial activation at 95°C for 15 min; 45 cycles of denaturation at 94°C for 15 s; annealing at 56°C for 30 s; extension at 72°C for 30 s, and a final extension at 72°C for 5 min followed by a melt step from 55°C to 95°C at 0.2°C/s.

### Human sample study population and tissue microarray (TMA) construction

Tissue samples originated from patients with primary breast carcinomas treated in the Edinburgh Breast Unit from 1999 to 2002. The study was approved by the Lothian Research Ethics Committee (08/S1101/41) and MREC (04/S0709/16). Ethical approval 08/S1101/41 authorises the distribution of FFPE samples and associated linked anonymised data from the Pathology archive (NHS Lothian) for research as they were surplus to diagnostic purposes. Axillary lymph node dissection was performed on all patients as part of surgery for large or high-grade invasive breast carcinomas. The extracted tissues were then embedded into a recipient paraffin block in a precisely spaced array pattern for further analysis. There were 136 primary and 105 nodal cancers available for statistical analysis after TMA construction, immunostaining, AQUAsition, and AQUAnalysis, including 65 paired samples. TMAs were constructed in biological triplicate. A second TMA series was then analysed for which outcome data were available, consisting of 546 breast cancers.

### Immunofluorescence and Automated QUantitative Analysis (AQUA) of protein expression

This assay was performed using optimized conditions for each target protein. IHC assays were performed to determine the optimum working conditions for phospho-STAT1(Tyr701), STAT1, phospho-STAT3(Ser727), STAT3, phospho-STAT5(Tyr694), and STAT5 antibodies (Figure S1 in File S1). The source of the STAT1 and STAT3 antibodies is described above while the STAT5 and pSTAT5(Tyr694) were both obtained from Cell Signaling. Briefly, sections were heat-treated under pressure in a microwave for 5 min in optimized antigen retrieval buffer and incubated with primary antibodies diluted to the optimal dilution in second primary antibody (mouse anti-cytokeratin, Invitrogen) diluted in Dako antibody diluents, either for 1 h at room temperature or overnight at 4°C. Sections were then incubated with secondary antibodies (including goat anti-mouse Alexa 555 antibody, Invitrogen) for 1 h at room temperature, following by target signal amplification diluents and the Cy5-tyramide for visualisation of target protein. Prolong Gold anti-fade reagent with DAPI (Invitrogen) nuclear visualisation media was applied to the coverslip. The immunofluorescence results were analysed with the Automated QUantitative Analysis (AQUA) system. After counterstaining and cover-slipping, the slides were imaged on the HistoRx PM-2000 instrument [28] utilizing an automated spot capturing system. Images were captured using the AQUAsition software and a 20x objective on the DAPI, CY3, and CY5 channels. For each immunofluorescence image, the AQUAnalysis software evaluated the quantity (in AQUA units = Au) of target protein expression (Cy5-tyramide signal) within the cytoplasm (identified by cytokeratin) and nuclei (4,6-diamidino-2-phenylindole, DAPI counterstaining). Target protein expression was scored only in invasive cancers and cores containing epithelium <5% of their total area were automatically excluded by the software to make sure that tumors were adequately represented for AQUA scoring [28].

### V250 Proteomic analysis

The proteomic microarray analysis was performed by Eurogentec (Eurogentec Ltd, Southampton, U.K.) which assessed the expression levels of 120 phosphorylated and non-phosphorylated signaling molecules that are known to be dysregulated in cancer. Protein lysates from the MCF7, MCF-7/LCC1, and MCF-7/LCC9 cell lines in the presence or absence of 1 nM 17Beta-estradiol (E_2_) were analysed using the V250 antibody array (Eurogentec Ltd, Southampton, U.K.). Each V250 array contains 240 antibodies that bind 120 signaling proteins in either their phosphorylated or non-phosphorylated forms. A full list of the antibodies used is shown in Table S1 in File S1. Binding of each antibody to its target results in an emission fluorescence, whose intensity is proportional to the level of the target protein. Each sample was run as six replicates. The intensity score for each phosphorylated protein was normalized by Eurogentec Ltd (Southampton, UK) to that of its non-phosphorylated counterpart.

### Statistical and bioinformatics analysis

The student's t-test was used for comparison of two independent samples. Spearman's rank correlation coefficient analysis was performed to detect associations from the immunofluorescence results. Data on 550 breast cancers from three publicly available gene expression datasets (GSE2990, GSE12093, GSE6532) were analysed (obtained from NCBI GEO). The Affymetrix U133A platform was used for GSE2990 and GSE12093 while the U133A, U133B, and U133 Plus 2.0 platforms were used for GSE6532. RMA normalisation was applied [29] and the datasets were integrated using ComBat to remove dataset-specific batch effects [30]. We used the software programme, X-Tile, to determine the optimal cutpoint while correcting for the use of minimum *P* statistics [31], which is known to inflate type I error when used incorrectly [32]. Two methods of statistical correction for the use of minimal *P* approach were utilised; the first calculation of a Monte Carlo *P*-value and for the second, the Miller-Siegmund minimal *P* correction [32]; the minimum *P*-value, Monte Carlo *P*-value, and Miller-Siegmund *P*-value were all required to be <0.05 for the cutpoint to be considered valid. Overall survival was subsequently assessed by Kaplan-Meier analysis with log-rank for determining statistical significance. The paired T-test was used for comparing target protein expression differences between the primary tumor and matched nodes. A p-value of <0.05 was considered statistically significant.

## Results

### STAT1 and STAT3 signaling pathways are differentially activated in endocrine sensitive and resistant breast cancer cell lines

In order to establish which pathways might influence estrogen signaling and endocrine therapy sensitivity and resistance, we initially carried out an unsupervised interrogation of biochemical signaling pathways using a phosphoprotein antibody array in MCF-7 sensitive and resistant breast cancer cell lines. The antibody array comprised 120 matched phospho- and non-phospho-antibodies designed to measure key epitopes within the majority of major growth factor, cell cycle, and DNA-damage response pathways (for a full list of targets see Table S1 in File S1). The ER-positive estrogen-dependent MCF-7 breast cancer cell line was compared with its estrogen-independent but tamoxifen and fulvestrant-sensitive cell line MCF-7/LCC1 [21] and the fully estrogen, tamoxifen and fulvestrant-resistant cell line MCF-7/LCC9 (LCC9; [22]). The most significantly differentially expressed targets are shown in Table 1 and the complete list is provided in Table S1 in File S1. Selected components of the STAT, MAPK, and NFκB pathways were both down- and up-regulated in MCF-7/LCC1 and MCF-7/LCC9 cell lines, while components of the mTOR and calcium signaling pathways were down-regulated and components of the PI3K, heat shock, and HGF signaling pathways were up-regulated in the resistant cell lines relative to MCF-7 expression. Since five of the top twenty differentially expressed phosphoprotein targets were components of the JAK/STAT pathway (STAT1, STAT3, TYK2, JAK1, JAK2) and STAT1 was the most differentially expressed total protein, we reasoned that STAT signaling might be a mediator of endocrine sensitivity and tamoxifen/fulvestrant resistance in breast cancer, prompting us to explore this association further.

**Table 1:** List of proteins and phospho-proteins significantly differentially expressed between LCC1 or LCC9 and parental MCF-7 cell lines.

Phospho Proteins	LCC1/MCF-7	LCC9/MCF-7
IKK alpha (Phospho-Thr23)	0.75	0.80
Rel (Phospho-Ser503)	0.72	0.98
Raf1 (Phospho-Ser259)	0.74	1.08
*STAT1 (Phospho-Ser701)*	1.19	1.18
p53 (Phospho-Ser6)	1.01	1.25
MEK1 (Phospho-Ser221)	1.03	1.26
PDK1 (Phospho-Ser241)	0.88	1.28
*STAT1 (Phospho-Ser727)*	*1.22*	*1.33*
HDAC8 (Phospho-Ser39)	1.00	1.34
JAK2 (Phospho-Tyr1007)	1.08	1.34
BAD (Phospho-Ser112)	1.04	1.35
Caveolin-1 (Phospho-Tyr14)	1.07	1.36
Beta-Catenin (Phospho-Thr41/Phospho-Ser45)	1.14	1.37
TYK2 (Phospho-Tyr1054)	1.12	1.43
Src (Phospho-Tyr418)	1.21	1.43
Met (Phospho-Tyr1349)	1.42	1.43
*STAT3 (Phospho-Ser727)*	*1.28*	*1.44*
JAK1 (Phospho-Tyr1022)	1.23	1.46
I-kappa-B-alpha (Phospho-Ser32/Phospho-Ser36)	1.45	1.52
HSP90B (Phospho-Ser254)	1.23	1.54
Akt (Phospho-Thr308)	1.32	1.59

non-Phospho Proteins	LCC1/MCF-7	LCC9/MCF-7
Elk-1 (Ab-383)	0.75	1.12
c-Jun (Ab-73)	1.17	1.25
I-kappa-B-epsilon (Ab-22)	1.04	1.26
ICAM-1 (Ab-512)	1.05	1.26
*STAT5A (Ab-694)*	*1.09*	*1.28*
p70 S6 Kinase (Ab-424)	1.00	1.28
14-3-3 Zeta (Ab-58)	1.07	1.28
NF kappa B-p105/p50 (Ab-337)	1.33	1.29
Raf1 (Ab-259)	1.00	1.29
p27Kip1 (Ab-10)	1.17	1.30
Beta-Catenin (Ab-41/45)	1.02	1.31
JAK2 (Ab-221)	0.98	1.31
JAK1 (Ab-1022)	1.09	1.31
CaMKII (Ab-286)	1.00	1.32
*STAT1 (Ab-701)*	*1.35*	*1.62*

The antibody array comprised 120 matched phospho- and non-phospho-antibodies designed to measure epitopes within major growth factor, cell cycle, and DNA-damage response pathways (for full list see Table S1 in File S1).

**Figure 1:**
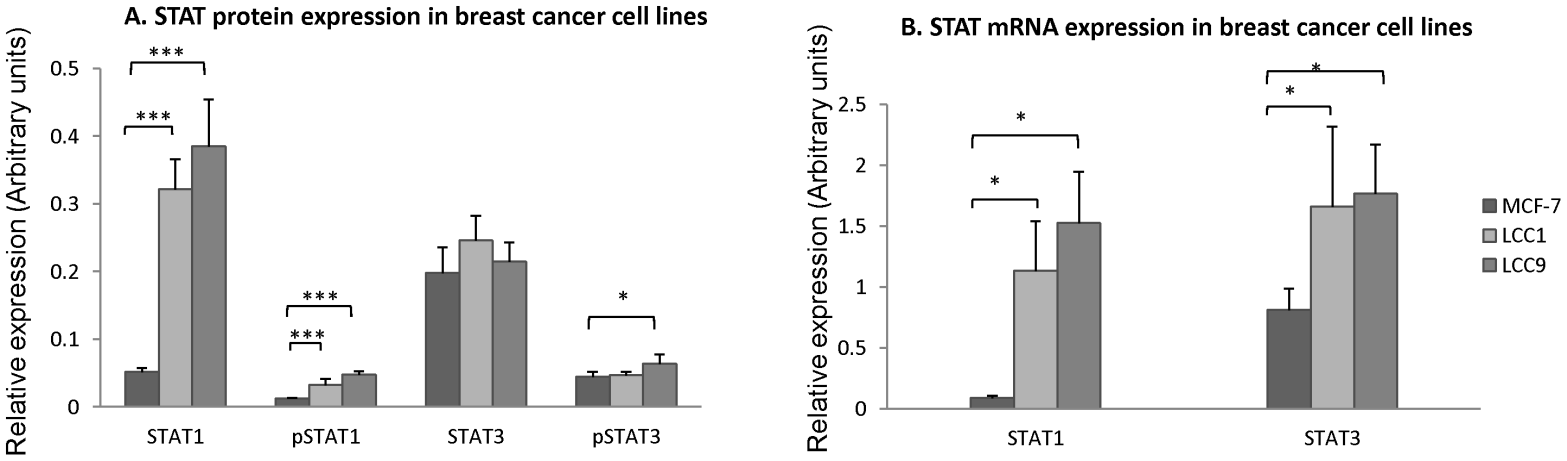
STAT protein (A) and mRNA (B) expression in the MCF-7, MCF-7/LCC1 (LCC1) and MCF-7/LCC9 (LCC9) breast cancer cell lines. A. MCF-7 cells were double charcoal-stripped for 48 h. Protein lysates were run on a 10% SDS-gel and membranes were probed with phospho-STAT1(Tyr 701), STAT1, phospho-STAT3 (Ser 727), or STAT3 primary antibodies (1∶1000). Column charts show the relative expression level of protein normalized with loading control (tubulin). Data are presented as relative mean Integrated Intensity (correlated with the fluorescence intensity of secondary antibody) ratios of target protein over tubulin +/− SEM from quadruplicate samples. Statistical significance noted for multiple comparision where *P<0.05, ***P<0.001 (student's t-test). B. mRNA expression of STAT was measured by two step real time PCR. Total RNA was extracted from cells charcoal stripped for 48 h. The cDNA was synthesised by reverse transcription, and real time PCR was performed as described in Materials and Methods. Relative expression of the target gene was normalized to that of Beta-actin. Results are presented as mean ±SD from triplicate samples.

We confirmed the results of the antibody array using semi-quantitative western blotting. Total STAT1 expression was increased in both the MCF-7/LCC1 cell line (∼6.4 fold, p<0.001) and MCF-7/LCC9 cell lines (∼7.4 fold, p<0.001) compared with the parental MCF-7 cell line. Similarly, phospho-STAT1 (Tyr701) expression was increased in both the MCF-7/LCC1 cell line and MCF-7/LCC9 cell lines compared with the MCF-7 cell line (Figure 1A) (Figure S2 in File S1). There was a statistically significant increase in phospho-STAT3 (Ser727) expression in the MCF-7/LCC9 cell line relative to the MCF-7 cell line (∼1.5 fold, p<0.05), while expression was similar between MCF-7 and MCF-7/LCC1 cells (not significant), and total STAT3 protein expression was the same in all cell lines.

Since total STAT1 expression levels differed between cell lines, we sought to establish whether this could be explained by underlying alterations in gene expression (Figure 1B). qRT-PCR analysis indicated that mRNA expression of STAT1 was increased in MCF-7/LCC1 cells (∼13 fold) and MCF-7/LCC9 cells (∼18 fold), compared with MCF-7 cells, while the difference in STAT3 mRNA expression was of lower magnitude with only a two-fold increase in expression in both MCF-7/LCC1 and MCF-7/LCC9 cells compared to MCF-7 cells, consistent with protein expression. Together these data show that STAT1 is differentially expressed in estrogen insensitive and tamoxifen/fulvestrant resistant cell lines at the mRNA, total protein, and activated protein level, and might therefore be a possible target for either single agent therapy or to overcome endocrine resistance.

### STAT inhibitors may be effective therapies, even in the endocrine-resistant setting

**Figure 2:**
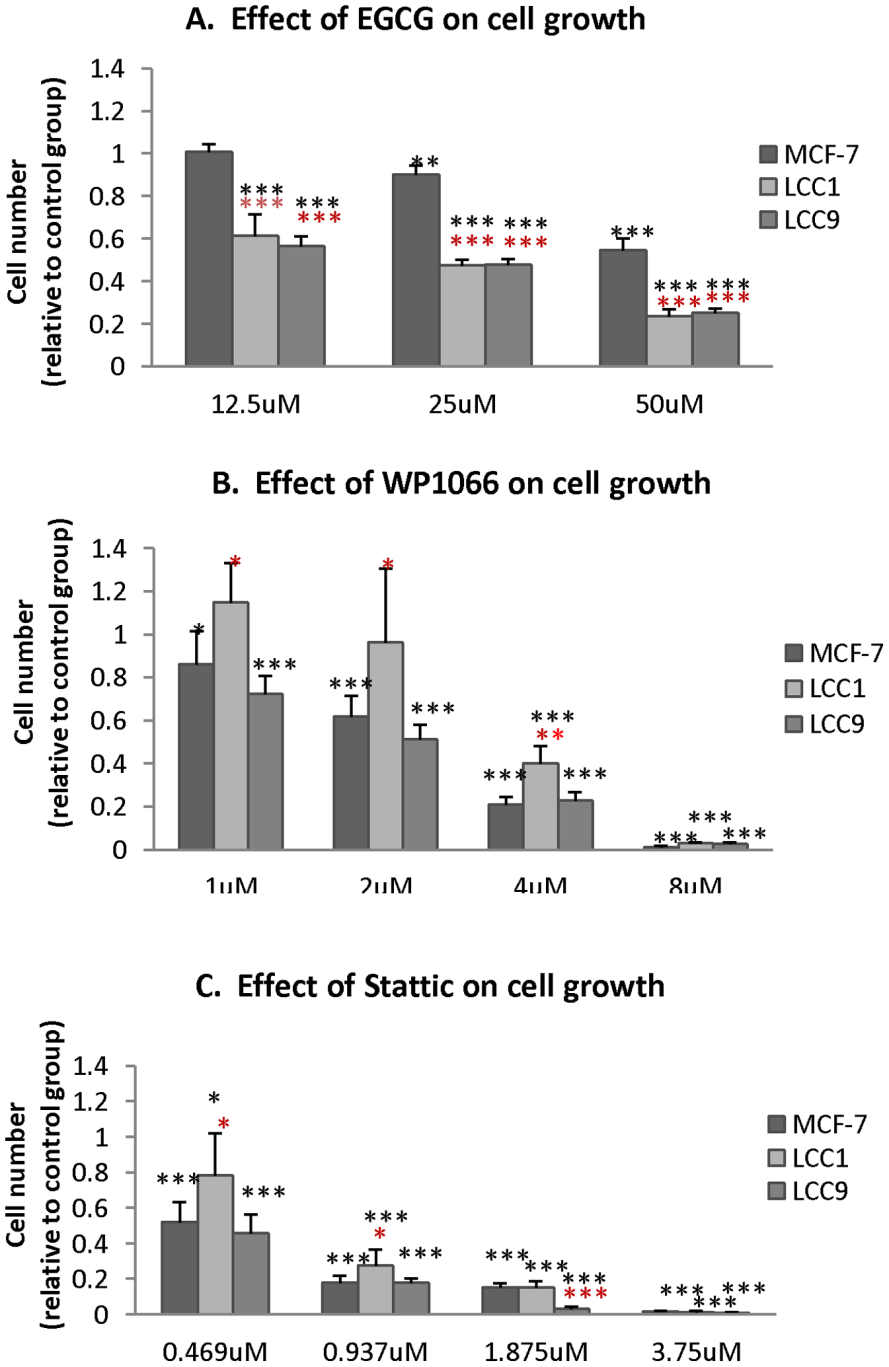
The effect of STAT inhibitors (A. EGCG, B. WP1066 and C. Stattic) on proliferation of Tamoxifen sensitive and resistant (MCF-7, MCF-7/LCC1 (LCC1), and MCF-7/LCC9 (LCC9)) cells. All cells were incubated in double charcoal-stripped medium for another 48 h after seeding and treated with or without inhibitor. O.D values were measured on Day 5. Data were plotted as a mean of O.D values +/− SD from 6 replicate samples. Asterisks represent significant changes between treatment and no treatment, while hashes represent differences between resistant cell lines and MCF7. Error bars are standard deviations. Statistical significance noted for treatment groups compared with untreated controls were ^#/^*P<0.05, ^##/^**P<0.01, ^###/^***P<0.001 (ANOVA followed by Tukey-Kramer).

We next investigated the relationship between STAT1 and STAT3 pathway activation and cell growth in the endocrine-resistant setting using the STAT1 inhibitor EGCG, and the STAT3 inhibitors Stattic and WP1066. Cell numbers were significantly reduced in response to the STAT1 inhibitor EGCG in the MCF-7/LCC1 and MCF-7/LCC9 cell lines compared to parental MCF7s (Figure 2), whereas both STAT3 inhibitors Stattic and WP1066 significantly reduced cell number in all three lines (Figure 2). STAT3 inhibition might therefore be a useful single agent or combination therapy in breast cancer while STAT1 inhibition may be more intimately associated with endocrine treatment failure and a useful therapeutic strategy to target resistance.

**Figure 3:**
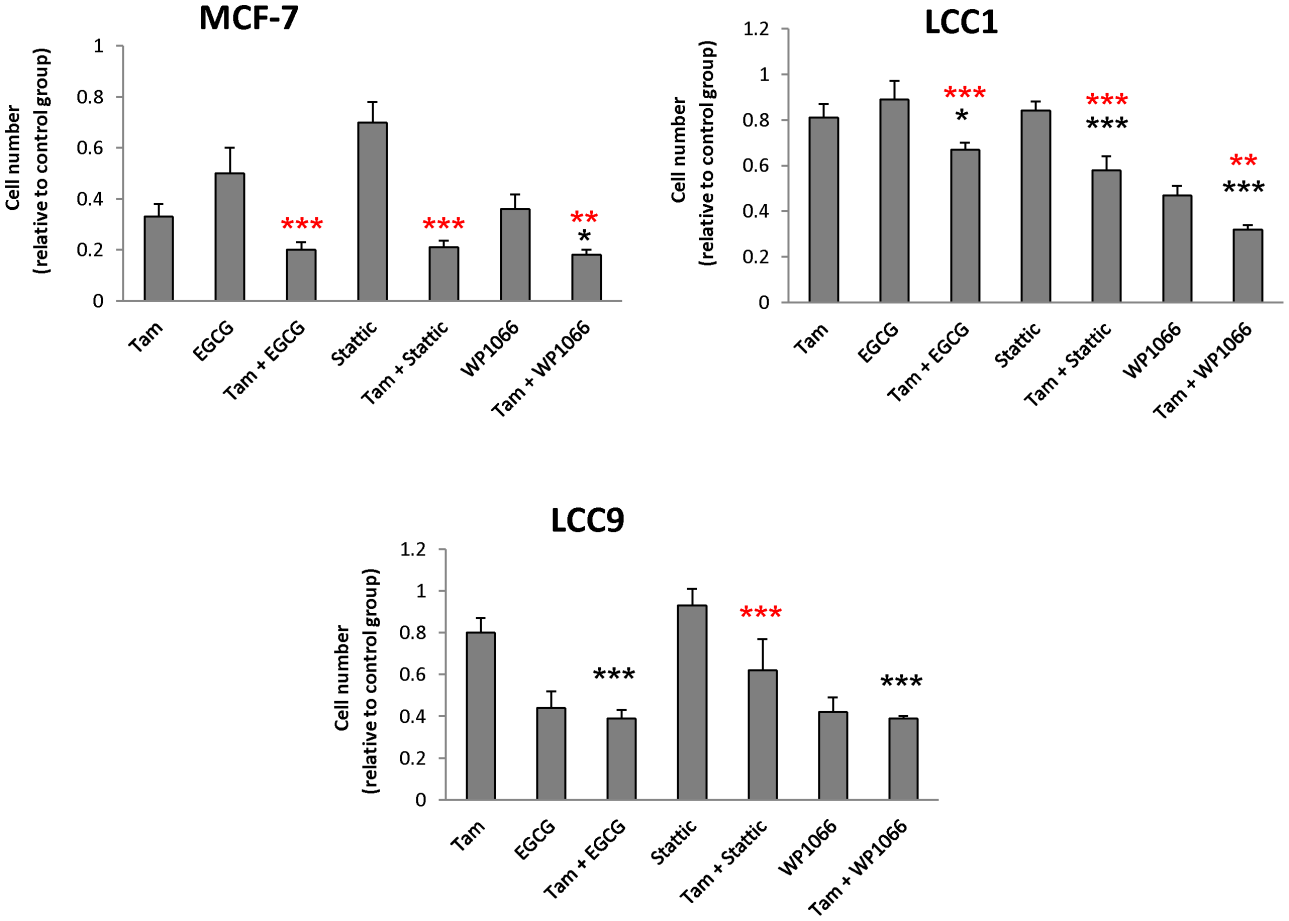
The effect of STAT inhibitors (EGCG, Stattic, or WP1066) combined with tamoxifen on MCF-7, MCF-7/LCC1 (LCC1), and MCF-7/LCC9 (LCC9) cells in the presence of estrogen. Cells were grown in charcoal-stripped serum, 48 h prior to treatment and were treated with control medium containing 1 nM E_2_, tamoxifen (1 µM)+E_2_ (1 nM), EGCG (25 µM)+E_2_ (1 nM), tamoxifen (1 µm)+EGCG (25 µm)+1 nM E_2_, Stattic (0.5 µM)+E_2_ (1 nM), tamoxifen (1 µM)+Stattic (0.5 µm)+E_2_ (1 nM), WP1066 (2 µM)+E_2_ (1 nM), tamoxifen (1 µM)+WP1066 (2 µM)+E_2_ (1 nM) for 5 days. O.D. values were measured on day 5. Data were plotted as a mean inhibition ratio of O.D. values over untreated control groups +/- SD from 4 replicate samples. Asterisks represent significant changes between combined treatment and the inhibitor alone, while hashes represent differences between combined treatment and tamoxifen treatment alone. ^#/^*P<0.05, ^##/^**P<0.01, ^###/^***P<0.001 (ANOVA followed by Tukey-Kramer).

We investigated the effect of STAT inhibition on the inhibitory effect of tamoxifen within the resistant cell line series. This experiment was undertaken in the presence of 1 nM E_2_. Under these conditions, the inhibitors had similar growth inhibitory effects against the 3 cell lines. The combination of EGCG, Stattic or WP1066 and tamoxifen caused a significant enhancement in inhibition of MCF-7 cells (p<0.05) compared with the STAT inhibitor alone (Figure 3). This combination treatment also produced an effect in the MCF-7/LCC1 cell line (Figure 3). In the MCF-7/LCC9 cell line, there was a minor increase in inhibition (Figure 3) for the combination treatment, however, a statistically significant enhancement was only noted for the Stattic group compared to single STAT inhibitor alone. STAT inhibitors therefore can provide a modest additive increase in efficacy over tamoxifen alone.

### Exploration of STAT expression in human breast cancer samples

**Figure 4:**
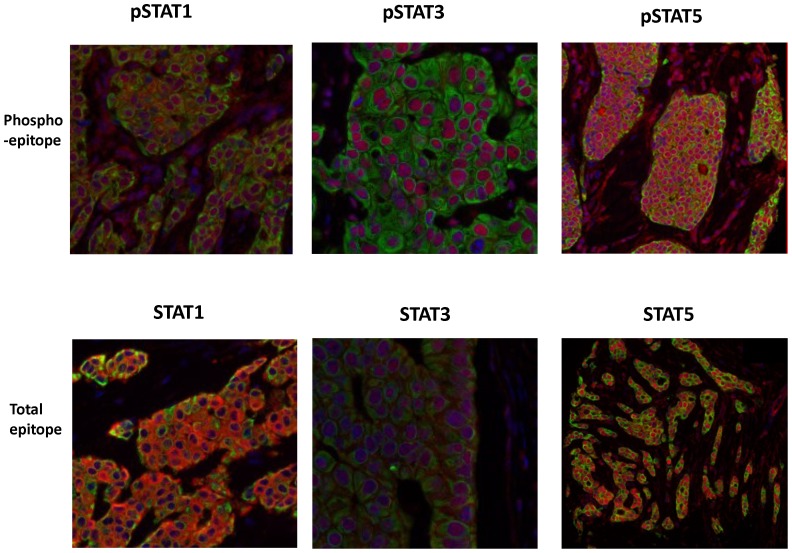
Comparison of STAT signaling in matched primary and metastatic tissue. Representative immunofluorescence images of phosphorylated and total STAT1, STAT3 and STAT5 in TMA cores. Blue  =  DAPI nuclear counterstain, green  =  cytokeratin tumor mask and red  =  target protein.

The above results suggest that STATs are implicated in breast cancer resistance. STAT isoform expression was measured in human breast cancer samples to investigate the associations between phosphorylated and total STAT protein expression and clinical parameters. We first analysed paired samples of primary breast cancers with their associated lymph node metastases to assess whether and how, STAT isoform expression changed with disease progression. A series of 136 primary and 105 nodal cancers were embedded within a TMA and immunofluorescence results were scored and analysed using Automated QUantitative Analysis (AQUA). Sixty-five of the primary cancers could be paired with their associated lymph node specimens. The AQUA scores represent the expression levels of target proteins in tumor nuclear or cytoplasmic compartments, using markers (DAPI or cytokeratin respectively) to help compartmentalise these areas. Representative immunofluorescence images for STAT protein expression in human breast cancer and associated nodal disease are shown in Figure 4. Expression of each STAT/phospho-STAT member was compared between primary cancer and nodal disease (Table 2). The mean expression of all the STATs and phospho-STATs, with the exception of phospho-STAT1(Tyr701) expression (and total STAT3 in the cytoplasm), were enhanced in their respective nodes relative to expression in the primary cancer. The most significant enhancements were exhibited in phospho-STAT3(Ser727) (∼1.5 fold) in nuclei, together with STAT1 (∼3.1 fold), phospho-STAT3(Ser727) (∼1.8 fold) and STAT5 (∼1.5 fold) in cytoplasm.

**Table 2:** Comparison of STAT and phospho-STAT expression between primary breast tumors (BC) and matched lymph node (LN) disease.

A. Expression in Nuclei					
		MAX	MIN	MEAN	P val
pSTAT1	BC	1375	358	727	0.02
	LN	1381	179	630	
pSTAT3	BC	1239	59	329	0.0006
	LN	1402	76	502	
pSTAT5	BC	172	36	89	0.0002
	LN	226	48	112	

B. Expression in cytoplasm					
		MAX	MIN	MEAN	P val
pSTAT1	BC	1005	316	604	0.051
	LN	974	173	541	
STAT1	BC	720	47	181	<0.0001
	LN	1683	119	568	
pSTATT3	BC	676	54	158	<0.0001
	LN	986	77	292	
STAT3	BC	2710	386	981	0.0034
	LN	1536	346	813	
pSTAT5	BC	71	23	46	<0.0001
	LN	163	31	68	
STATT5	BC	810	157	303	<0.0001
	LN	1335	143	502	

Immunofluorescence data were analysed using AQUA. The maximum, minimum, and mean AQUA values of each protein associated with the expression level are listed for both nuclear and cytoplasmic expression. A paired t-test was performed to compare whether the difference of expression between primary tumors and nodal disease was significant, and p values are shown in the tables.

Next, associations between different STAT isoforms in the same sample were analysed and associated with previously obtained data for other markers (ER, PR, HER2, CK5/6, and EGFR) on these samples. The Spearman's rank correlation coefficient was measured for every combination of these STATs or phospho-STATs expression level in both primary tumors and paired nodes (Table S2 and S3 in File S1). All phospho-STATs were highly significantly correlated (nearly all p-values <0.0001) with each other in both primary and nodal tumors, among which cytoplasmic phospho-STAT1(Tyr701) - nuclear phospho-STAT1(Tyr701), cytoplasmic phospho-STAT3(Ser727) – nuclear phospho-STAT3(Ser727), and cytoplasmic phospho-STAT5(Tyr694) – nuclear phospho-STAT5(Tyr694) had significantly higher correlation coefficients with r values from 0.74 to 0.95. For the non-phosphorylated STATs, significant correlations also existed within each pair with the highest r values from 0.69 to 0.72, except the STAT1 group in the nodal data. The associations between the STATs and other markers were then analysed. Of particular note, ER expression was strongly associated with cytoplasmic expression of STAT1 in primary breast cancers (p = 0.0003) and in the associated nodes (p = 0.005). Expression of PR was also very strongly associated with STAT1 in the primary breast cancers (p<0.0001) although not in nodal disease. Inverse correlations between EGF receptor expression and both total STAT1 and phospho-STAT1(Tyr701) were observed in primary breast cancers (p<0.05) with a marked inverse association for total STAT1 in paired nodal breast cancers (p<0.0001) (Table S3 in File S1)

**Figure 5:**
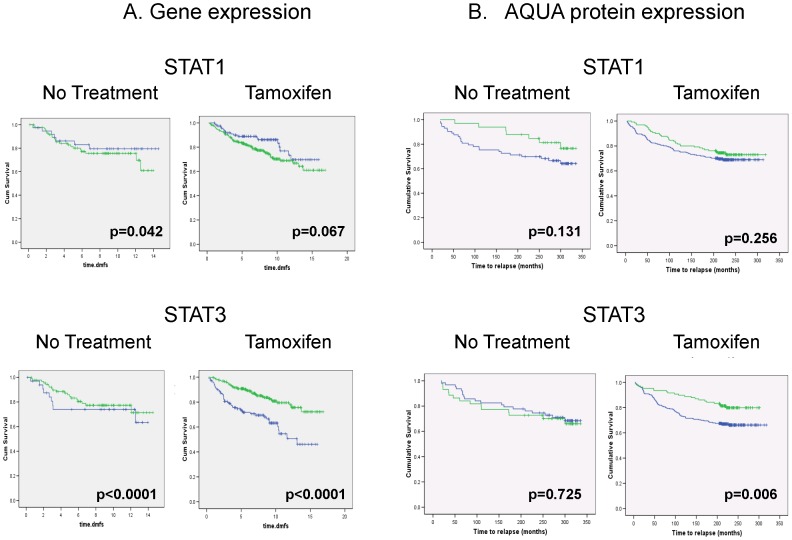
STAT expression and prognosis. Gene (A) and protein (B) expression of STAT1 and STAT3 in primary breast cancer (blue = low, green = high). Optimal cut-points were determined using the x-tile program while correcting for the use of minimum P statistics (31). Low STAT3 expression is associated with very poor outcomes for patients treated with tamoxifen (protein expression STAT3 high (78% fifteen-year survival) vs. STAT3 low (68% five year survival).

Expression levels of STAT1 and STAT3 transcript were then analyzed in 550 breast cancers from publicly available gene expression datasets (GSE2990, GSE12093, GSE6532) (Figure 5A), and protein expression was measured in an independent cohort of 546 primary breast cancers (Figure 5B). Low expression of STAT3 was associated with very poor distant metastasis free survival (DMFS) in patients treated with tamoxifen when measured either by gene or protein expression (Figure 5; protein expression RR 1.58: 95% CIs 1.1-2.2, p = 0.006). Although there were trends to poorer outcome for tumors with high expression of STAT1, this was only of borderline significance. STAT3 expression may therefore be predictive of outcome in tamoxifen-treated patients.

## Discussion

The STAT family of molecules are key regulators of normal cellular physiology and have important roles in many pathological conditions [1]–[5]. Their functions in endocrine-sensitive and resistant breast cancer are still poorly defined and the published literature indicates complex roles, particularly for STAT1 and STAT3. Previous investigations into the roles of the STAT family in endocrine-resistant breast cancer, especially STAT3 and STAT5, have shown associations with tumorigenicity, cell-cycle progression, cell survival, transformation, and angiogenesis [10]. Furthermore, increasing lines of evidence indicated their involvement in the oncogenesis of breast cancer and in resistance to endocrine therapy; however, there have been few studies on STAT1 in breast cancer, particularly relating to endocrine treatment resistance. STAT1 expression has previously been shown to be associated with resistance to DNA damage and genotoxic agents which is likely to produce associations with poor outcome [33], [34]. In long-term estrogen exposed MCF-7 cells, STAT1 expression levels are increased and this again has been suggested to associate with increased resistance to radiotherapy and chemotherapy [35]. STAT1 and STAT3 activation have previously been suggested to be associated with tamoxifen resistance in MaCa 3366/TAM although expression was not changed in this model system [36]. To evaluate the roles of STAT1 and STAT3 further, we used cell line models to study functional responses in endocrine sensitive and resistant breast cancer cells and then primary breast cancer samples to evaluate the associations between expression levels and clinical outcome.

In this study, a 3-stage MCF-7 cell line derived model (MCF-7, MCF-7/LCC1 and MCF-7/LCC9) [21], [22] was used to mimic the clinical development of endocrine resistance in breast cancer. The initial proteomic analysis indicated that among some of the major signaling pathways known to be key in cancer cells, activated STAT1 and STAT3, were differentially expressed and therefore potentially interesting signaling candidates in resistant cells. These differences were first confirmed by protein and mRNA analysis. The increased expression of STAT1, phospho-STAT1(Tyr701), and phospho-STAT3(Ser727) in resistant cells compared with sensitive ones suggested possible roles for STAT1 and STAT3 in the development of endocrine resistance. Inhibition of both STAT1 and STAT3 using available small molecule inhibitors produced growth inhibition. The anti-proliferative effect of EGCG in the resistant lines suggested that STAT1 might be important in the development of resistant breast cancer. EGCG has been shown to have a synergistic action in combination with anti-estrogen strategies in ER-negative breast cancer cells [37]–[39] and can attenuate growth and invasion in other tamoxifen-resistant breast cancer cell line models [40]. Our results indicate that it may also have potential value in endocrine-resistant disease.

Expression levels of both the total and phospho-activated STAT isoform proteins show complex associations with stage and outcome in clinical breast cancer samples as evidenced by the varying results in the literature. This variability is likely to be due in part to a different balance of subgroups of breast cancers being investigated in different studies. Our emphasis here was on ER-positive breast cancer and it is likely that results of ER-positive disease treated with endocrine therapy may well differ from population studies with a large percentage of ER-negative disease patients treated with chemotherapy. Our analysis of clinical breast cancers indicated that increased total STAT3 expression was associated with favourable outcome whether examined at the mRNA or protein level. Our data, based on two independent cohorts using two different, but complementary technologies, suggest that STAT3 is predictive for outcome in tamoxifen-treated patients, consistent with its putative role in hormonally-driven tumors. This is consistent with the studies of Sonnenblich et al. [16] and Dollhed-Fillhard et al. [15] which demonstrated improved survival in phospho-STAT3(Tyr705) expressing breast cancers. Sonnenblich et al. [16] pointed to the study by Dien et al. [41] showing that STAT3 upregulates tissue inhibitor of metalloproteinase-1 (TIMP1) expression, which decreases invasiveness of breast cancer cells. Of interest, STAT3 phosphorylation has previously been shown to be associated with local regional node involvement but not with distant metastases [42]. Our data suggest increased levels in local lymph nodes relative to the primary breast cancers but this may not be the same in distant metastasis. Total STAT1 expression demonstrated borderline association with prognosis, with higher mRNA expression being associated with poorer outcome. This was however, not supported by the protein expression data. The results of studies investigating the prognostic and predictive significance of STAT1 expression are variable. A microarray study evaluated STAT1 expression in a large series of breast cancers and expression was observed in 21% of 923 breast cancers with its presence being associated with poor survival [12]. However in a smaller study of 102 breast cancers, no association with outcome was observed [43]. In a study of 47 premenopausal and 118 postmenopausal breast cancer patients, tumor expression of phosphorylated STAT1(Tyr 701) was reported to correlate with poor survival in premenopausal women, but not in postmenopausal women [13]. In contrast, in a study reported by Widschwendter et al., increased STAT1 activation and phosphorylation were associated with favourable outcome [14]. Both studies investigated phospho-STAT1(Tyr701), but used different antibodies perhaps contributing to the different results. Therefore, while associations are being identified between STAT1 expression and outcome, the results are conflicting. It is interesting that STAT5 is also reported to be associated with favourable outcome in breast cancer for both total STAT5 [44] and nuclear phospho-STAT5 [45], [46] yet, like STAT3, this is considered to have a pro-oncogenic role in early disease development [8], [9].

It is feasible that STAT1 and STAT3 may have differing roles in different breast cancer subgroups dependent on whether they are hormonally or growth factor-driven. Hence, functionality may vary between endocrine-sensitive and endocrine-resistant ER-positive breast cancer and ER-negative disease. Associations between STAT1 and ER expression have been reported [13], and in our study, we observed highly significant correlations between STAT1 and ER expression and an inverse correlation with EGF receptor expression. Therefore, if ER-positive (luminal A type) cancers have a higher level of expression of STAT1 than other breast cancer types [19], they may represent the best target group for STAT1-targeted approaches.

In conclusion, these results indicate that STAT1 expression and activation can be increased in endocrine-resistant breast cancer and STAT1 inhibitors may be effective in resistant cells. In clinical samples, STAT isoform expression can increase between primary cancer and their matched lymph node metastases, suggesting a link with disease progression. These results indicate that STAT1 may represent a viable target in endocrine-resistant disease and merit further studies with STAT1 targeted inhibitors.

## Supporting Information

File S1 **Figures S1–S2 and Tables S1–S3.** Figure S1. Examples of immunohistochemistry staining for STAT1(a), phospho-STAT1(Tyr701) (b), STAT3 (c), phospho-STAT3(Ser727) (d), STAT5 (e), phospho-STAT5(Tyr694) (f). Figure S2. Western blots of STAT1, p-STAT1, STAT3 and p-STAT3 for the MCF7, LCC1 and LCC9 cell lines. Quadruplicate samples are shown. Tubulin was used as loading control. Table S1. Full list of targets in V250 proteomic antibody array. Table S2. Spearman's rank correlation coefficient analysis for STATs/pSTATs, ER, PR, HER2, CK5/6, and EGFR expression in primary breast tumors. The first row of each compared pair showed p value (no correlation as null hypothesis), and correlation coefficient was listed in the second row underneath. Numbers in bold represents high correlation with p value<0.05. Table S3. Spearman's rank correlation coefficient analysis for STATs/pSTATs, ER, PR, HER2, CK5/6, and EGFR expression in paired lymph nodes. The first row of each compared pair showed p value (no correlation as null hypothesis), and correlation coefficient was listed in the second row underneath. Numbers in bold represents high correlation with p value<0.05.
(DOCX)
