# Supplementary material for: Increased STAT1 Signaling in Endocrine-Resistant Breast Cancer
Source: PLoS One. 2014 Apr 11;9(4):e94226. doi: 10.1371/journal.pone.0094226 (PMC3984130; doi:10.1371/journal.pone.0094226)
Supplement: File S1 — Figures S1–S2 and Tables S1–S3. Figure S1. Examples of immunohistochemistry staining for STAT1(a), phospho-STAT1(Tyr701) (b), STAT3 (c), phospho-STAT3(Ser727) (d), STAT5 (e), phospho-STAT5(Tyr694) (f). Figure S2. Western blots of STAT1, p-STAT1, STAT3 and p-STAT3 for the MCF7, LCC1 and LCC9 cell lines. Quadruplicate samples are shown. Tubulin was used as loading control. Table S1. Full list of targets in V250 proteomic antibody array. Table S2. Spearman's rank correlation coefficient analysis for STATs/pSTATs, ER, PR, HER2, CK5/6, and EGFR expression in primary breast tumors. The first row of each compared pair showed p value (no correlation as null hypothesis), and correlation coefficient was listed in the second row underneath. Numbers in bold represents high correlation with p value<0.05. Table S3. Spearman's rank correlation coefficient analysis for STATs/pSTATs, ER, PR, HER2, CK5/6, and EGFR expression in paired lymph nodes. The first row of each compared pair showed p value (no correlation as null hypothesis), and correlation coefficient was listed in the second row underneath. Numbers in bold represents high correlation with p value<0.05. (DOCX) [file pone.0094226.s001.docx]

**Figure S1. Examples of immunohistochemistry staining for STAT1 (a), pSTAT1 (b), STAT3 (c), pSTAT3 (d), STAT5 (e), pSTAT5 (f).**


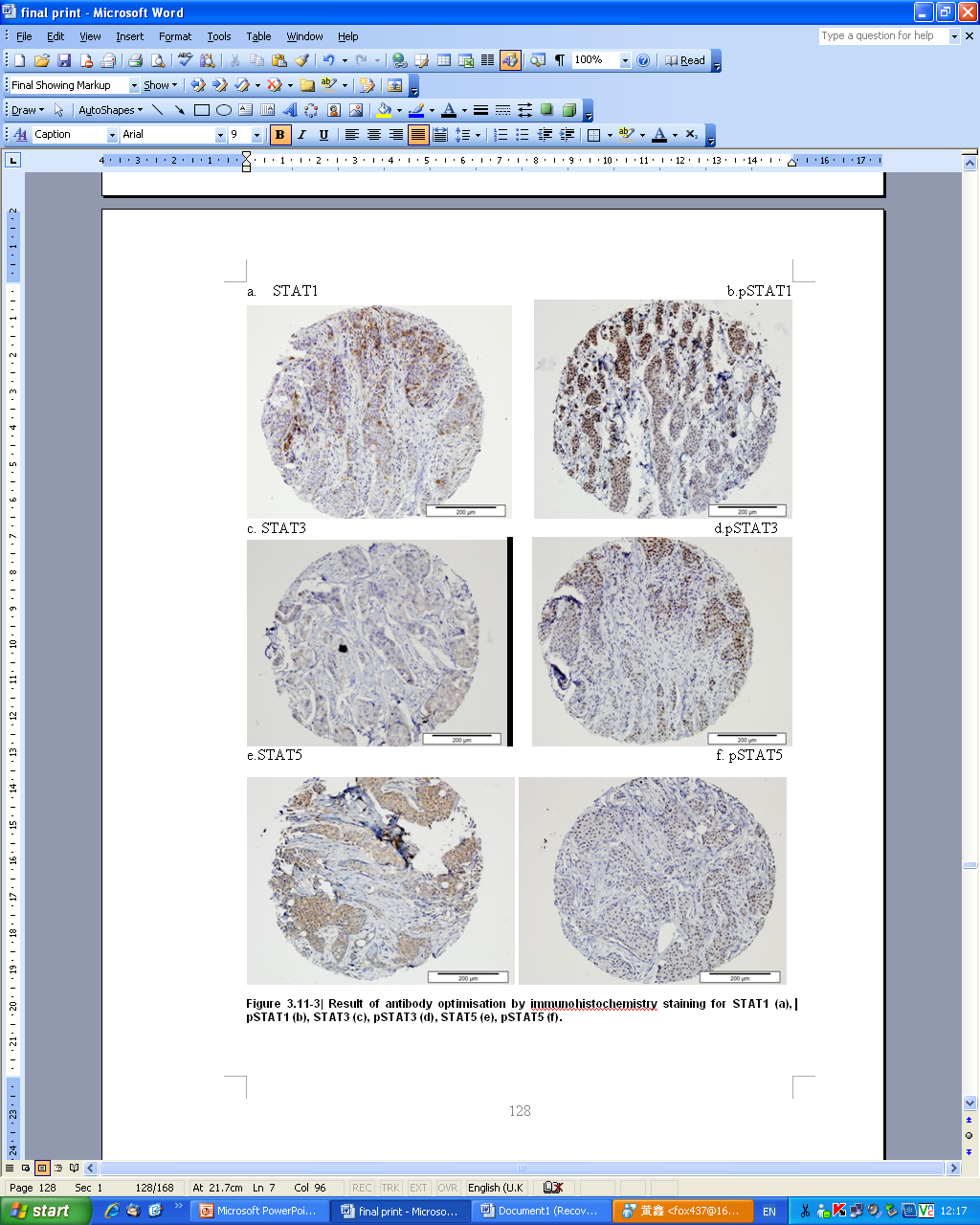


**Figure S2. Western blots of Stat1, p-Stat1, Stat3 and p-Stat3 for the MCF7, LCC1 and LCC9 cell lines.** Quadruplicate samples are shown. Tubulin was used as loading control.

**
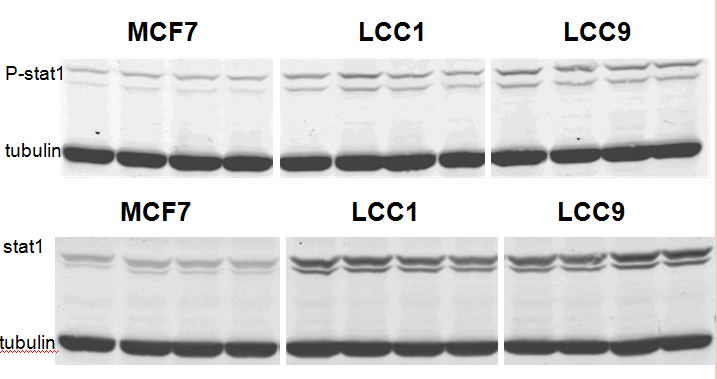
**
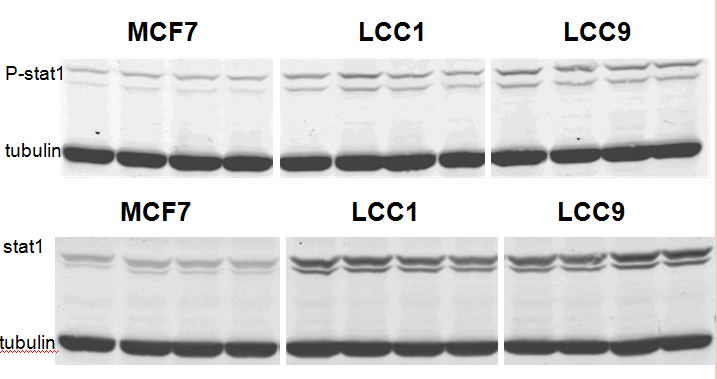


**
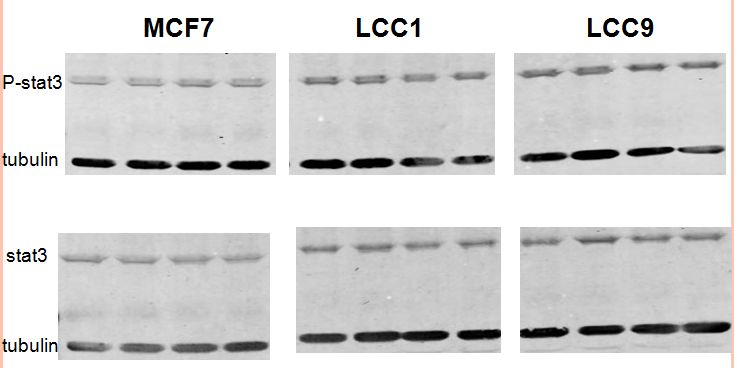
**

**
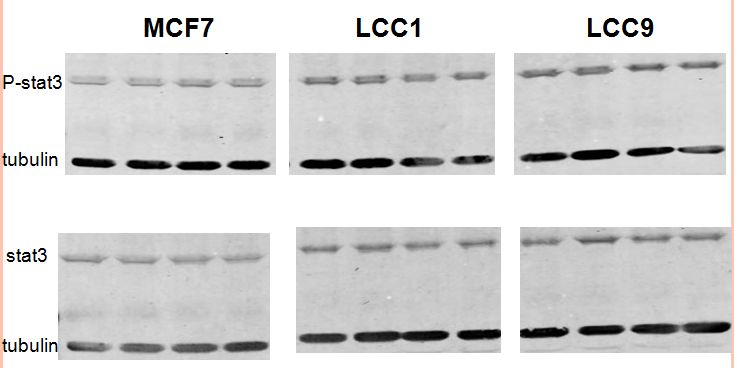
**

| 14-3-3 Zeta(Ab-58) | Beta-Catenin(Phospho-Ser33) | Chk2(Phospho-Thr68) | FAK(Phospho-Tyr861) | IGF-1R (Ab-1161) | MEK1(Ab-217) | NF kappa B-p105/p50(Ab-337) | p70 S6 Kinase (Phospho-Ser424) | STAT1(Ab-701) |
| --- | --- | --- | --- | --- | --- | --- | --- | --- |
| 14-3-3 Zeta(Phospho-Ser58) | Beta-Catenin(Phospho-Ser37) | c-Jun (Phospho-Thr239) | FAK(Phospho-Tyr925) | IGF-1R (Phospho-Tyr1161) | MEK1(Ab-221) | NF kappa B-p105/p50(Ab-893) | PDGF Receptor Beta(Ab-751) | STAT1(Ab-727) |
| 4E-BP1(Ab-36) | Beta-Catenin(Phospho-Thr41/Phospho-Ser45 | c-Jun(Ab-239) | FGF Receptor 1(Ab-154) | I-kappa-B-alpha(Ab-32/36) | MEK1(Ab-291) | NF kappa B-p105/p50(Ab-907) | PDGF Receptor beta(Phospho-Tyr751) | STAT1(Phospho-Ser727) |
| 4E-BP1(Phospho-Thr36) | BRCA1(Ab-1423) | c-Jun(Ab-243) | FGF Receptor 1(Phospho-Tyr154) | I-kappa-B-alpha(Phospho-Ser32/Phospho-Ser36 | MEK1(Phospho-Ser217) | NF kappa B-p105/p50(Phospho-Ser893) | PDK1(Ab-241) | STAT1(Phospho-Tyr701) |
| Akt(Ab-308) | BRCA1(Ab-1524) | c-Jun(Ab-73) | FKHR(Ab-256) | I-kappaB-alpha(Phospho-Tyr42) | MEK1(Phospho-Ser221) | NF kappa B-p105/p50(Phospho-Ser907) | PDK1(Phospho-Ser241) | STAT3(Ab-705) |
| Akt(Ab-473) | BRCA1(Phospho-Ser1423) | c-Jun(Phospho-Ser243) | FKHR(Phospho-Ser256) | I-kappa-B-beta(Phospho-Ser23) | MEK1(Phospho-Thr291) | NF kappa B-p105/p50(Phospho-Ser932) | PTEN(Ab-380/382/383) | STAT3(Ab-727) |
| Akt(Phospho-Ser473) | BRCA1(Phospho-Ser1524) | c-Jun(Phospho-Ser73) | GAPDH | I-kappa-B-epsilon(Ab-22) | MEK-2(Ab-394) | NF kappa B-p65(Ab-254) | PTEN(Phospho-Ser380/Phospho-Thr382/Phosp-Thr383 | STAT3(Phospho-Ser727) |
| Akt(Phospho-Thr308) | CaMKII (Ab-286) | c-Kit(Ab-721) | GSK3-alpha(Ab-21) | I-kappa-B-epsilon(Phospho-Ser22) | MEK-2(Phospho-Thr394) | NF kappa B-p65(Ab-529) | Pyk2(Ab-402) | STAT3(Phospho-Tyr705) |
| Akt2(Ab-474) | CaMKII (Phospho-Thr286) | c-Kit(Phospho-Tyr721) | GSK3-alpha(Phospho-Ser21) | IKK alpha(Ab-23) | Met(Ab-1349) | NF kappa B-p65(Phospho-Ser529) | Pyk2(Phospho-Tyr402) | STAT4(Ab-693) |
| Akt2(Phospho-Ser474) | Caveolin-1(Ab-14) | CREB(Ab-133) | GSK3-beta(Ab-9) | IKK alpha(Phospho-Thr23) | Met(Phospho-Tyr1349) | NF kappa B-p65(Phospho-Thr254) | Rac1/cdc42(Ab-71) | STAT4(Phospho-Tyr693) |
| AMPK1(Ab-174) | Caveolin-1(Phospho-Tyr14) | CREB(Phospho-Ser133) | GSK3-beta(Phospho-Ser9) | Integrin beta-3(Ab-773) | MKK3(Ab-189) | p21Cip1(Ab-145) | Rac1/cdc42(Phospho-Ser71) | STAT5A (Ab-694) |
| AMPK1(Phospho-Thr174) | CDC2(Ab-15) | CrkII(Ab-221) | HDAC8(Ab-39) | Integrin beta-3(Ab-785) | MKK3(Phospho-Ser189) | p21Cip1(Phospho-Thr145) | Raf1(Ab-259) | STAT5A (Ab-780) |
| ATM(Ab-1981) | CDC2(Phospho-Tyr15) | CrkII(Phospho-Tyr221) | HDAC8(Phospho-Ser39) | Integrin beta-3(Phospho-Tyr773) | MSK1(Ab-376) | p27Kip1(Ab-10) | Raf1(Phospho-Ser259) | STAT5A (Phospho-Ser780) |
| BAD(Ab-112) | cdc25A (Ab-75) | eEF2K(Ab-366) | HER2(Ab-877) | Integrin beta-3(Phospho-Tyr785) | MSK1(Phospho-Ser376) | p27Kip1(Ab-187) | Rb(Ab-780) | STAT5A (Phospho-Tyr694) |
| BAD(Ab-136) | cdc25A (Phospho-Ser75) | eEF2K(Phospho-Ser366) | HER2(Phospho-Tyr877) | JAK1(Ab-1022) | mTOR(Ab-2448) | p27Kip1(Phospho-Ser10) | Rb(Phospho-Ser780) | STAT6(Ab-641) |
| BAD(Ab-155) | cdc25C(Ab-216) | EGFR(Ab-1110) | Histone H2A.X(Ab-139) | JAK1(Phospho-Tyr1022) | mTOR(Phospho-Ser2448) | p27Kip1(Phospho-Thr187) | Rel(Ab-503) | STAT6(Ab-645) |
| BAD(Phospho-Ser112) | cdc25C(Phospho-Ser216) | EGFR(Phospho-Tyr1110) | Histone H2A.X(Phospho-Ser139) | JAK2(Ab-1007) | Myc(Ab-358) | P38 MAPK(Ab-182) | Rel(Phospho-Ser503) | STAT6(Phospho-Thr645) |
| BAD(Phospho-Ser136) | CDK2(Ab-160) | eIF2 alpha(Ab-51) | HSF1(Ab-303) | JAK2(Ab-221) | Myc(Ab-373) | P38 MAPK(Phospho-Thr180) | SAPK/JNK(Ab-183) | STAT6(Phospho-Tyr641) |
| BAD(Phospho-Ser155) | CDK2(Phospho-Thr160) | eIF2 alpha(Phospho-Ser51) | HSF1(Phospho-Ser303) | JAK2(Phospho-Tyr1007) | Myc(Ab-58) | P38 MAPK(Phospho-Tyr182) | SAPK/JNK(Phospho-Thr183) | Tau(Ab-404) |
| BCL-2(Ab-56) | Chk1(Ab-280) | elF4E(Ab-209) | HSP27(Ab-15) | JAK2(Phospho-Tyr221) | Myc(Ab-62) | p44/42 MAP Kinase(Ab-202) | Shc(Ab-349) | Tau(Phospho-Ser404) |
| BCL-2(Ab-70) | Chk1(Ab-317) | elF4E(Phospho-Ser209) | HSP27(Ab-78) | JunB(Ab-259) | Myc(Phospho-Ser373) | p44/42 MAP Kinase(Ab-204) | Shc(Phospho-Tyr349) | Trk B(Ab-515) |
| BCL-2(Phospho-Ser70) | Chk1(Ab-345) | Elk-1(Ab-383) | HSP27(Phospho-Ser15) | JunB(Ab-79) | Myc(Phospho-Ser62) | p44/42 MAP Kinase(Phospho-Thr202) | SHP-2(Ab-580) | Trk B(Phospho-Tyr515) |
| BCL-2(Phospho-Thr56) | Chk1(Phospho-Ser280) | Elk-1(Phospho-Ser383) | HSP27(Phospho-Ser78) | JunB(Phospho-Ser259) | Myc(Phospho-Thr358) | p44/42 MAP Kinase(Phospho-Tyr204) | SHP-2(Phospho-Tyr580) | TYK2(Ab-1054) |
| BCL-XL(Ab-62) | Chk1(Phospho-Ser317) | Estrogen Receptor-alpha (Ab-167) | HSP90B(Ab-254) | JunB(Phospho-Ser79) | Myc(Phospho-Thr58) | p53(Ab-315) | Smad3(Phospho-Ser425) | TYK2(Phospho-Tyr1054) |
| BCL-XL(Phospho-Ser62) | Chk1(Phospho-Ser345) | Estrogen Receptor-alpha (Phospho-Ser167) | HSP90B(Phospho-Ser254) | JunD(Ab-255) | NF kappa B-p100/p52(Ab-865) | p53(Ab-6) | Src(Ab-418) | VEGFR2(Ab-951) |
| Beta actin | Chk2(Ab-516) | FAK(Ab-397) | I kappaB-alpha(Ab-42) | JunD(Phospho-Ser255) | NF kappa B-p100/p52(Ab-869) | p53(Phospho-Ser315) | Src(Ab-529) | VEGFR2(Phospho-Tyr951) |
| Beta-Catenin(Ab-37) | Chk2(Ab-68) | FAK(Ab-861) | ICAM-1(Ab-512) | Keratin 18(Ab-33) | NF kappa B-p100/p52(Phospho-Ser869) | p53(Phospho-Ser6) | Src(Phospho-Tyr418) |  |
| Beta-Catenin(Ab-41/45) | Chk2(Phospho-Ser516) | FAK(Ab-925) | ICAM-1(Phospho-Tyr512) | Keratin 18(Phospho-Ser33) | NF kappa B-p105/p50(Ab-337) | p70 S6 Kinase (Ab-424) | Src(Phospho-Tyr529) |  |

**Table S1: Full list of targets in V250 proteomic antibody array**

Table S2 Spearman's rank correlation coefficient analysis for STATs/pSTATs, ER, PR, HER2, CK5/6, and EGFR expression in primary breast tumors. The first row of each compared pair showed p value (no correlation as null hypothesis), and correlation coefficient was listed in the second row underneath. Numbers in bold represents high correlation with p value < 0.05.


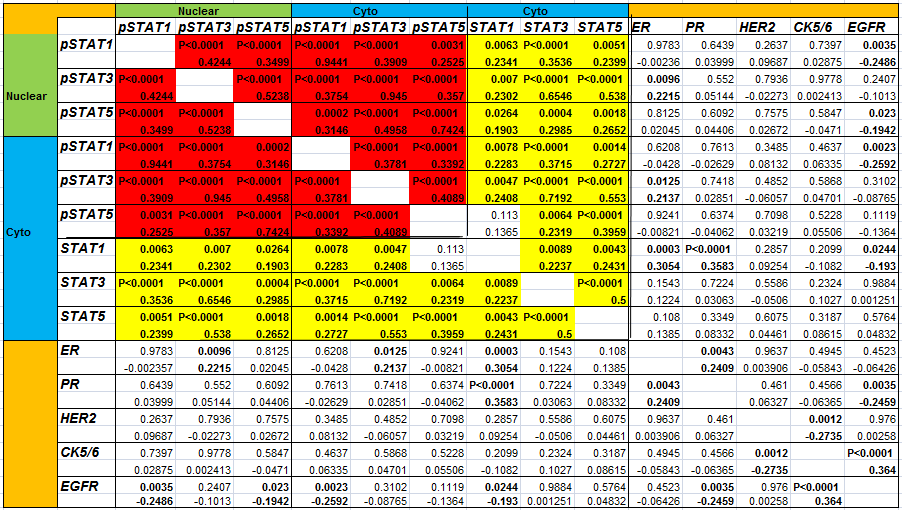


Table S3 Spearman's rank correlation coefficient analysis for STATs/pSTATs, ER, PR, HER2, CK5/6, and EGFR expression in paired lymph nodes. The first row of each compared pair showed p value (no correlation as null hypothesis), and correlation coefficient was listed in the second row underneath. Numbers in bold represents high correlation with p value < 0.05.

**
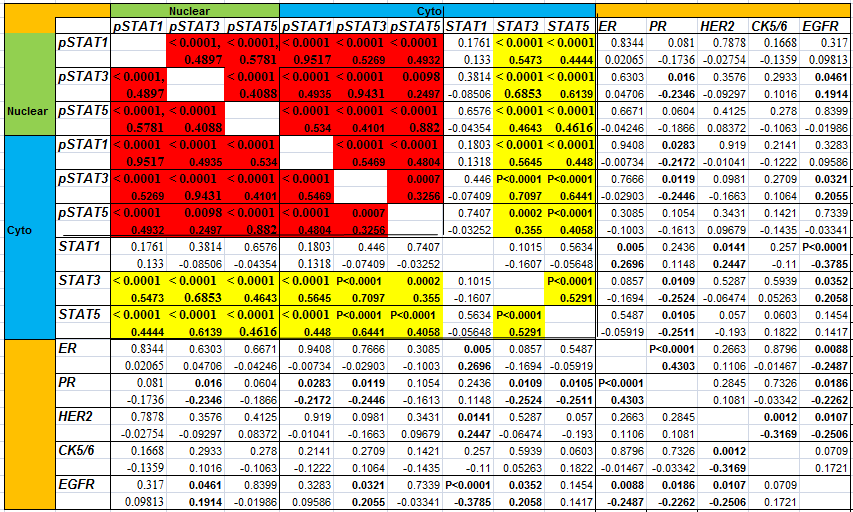
**
